# Gut Anaerobes Capable of Chicken Caecum Colonisation

**DOI:** 10.3390/microorganisms7120597

**Published:** 2019-11-21

**Authors:** Tereza Kubasova, Miloslava Kollarcikova, Magdalena Crhanova, Daniela Karasova, Darina Cejkova, Alena Sebkova, Jitka Matiasovicova, Marcela Faldynova, Frantisek Sisak, Vladimir Babak, Alexandra Pokorna, Alois Cizek, Ivan Rychlik

**Affiliations:** 1 Veterinary Research Institute, 621 00 Brno, Czech Republic; kubasova@vri.cz (T.K.); kollarcikova@vri.cz (M.K.); crhanova@vri.cz (M.C.); karasova@vri.cz (D.K.); cejkova@vri.cz (D.C.); asebkova@vri.cz (A.S.); matiasovicova@vri.cz (J.M.); faldynova@vri.cz (M.F.); sisak@vri.cz (F.S.); babak@vri.cz (V.B.); 2 Department of Infectious Diseases and Microbiology, Faculty of Veterinary Medicine, University of Veterinary and Pharmaceutical Sciences Brno, 612 42 Brno, Czech Republic; pokornaa@vfu.cz (A.P.) cizeka@vfu.cz (A.C.); 3 Central European Institute of Technology (CEITEC), University of Veterinary and Pharmaceutical Sciences Brno, 612 42 Brno, Czech Republic

**Keywords:** caecum, chicken, oral inoculation, colonisation, *Salmonella*, Bacteroidetes, Firmicutes

## Abstract

Chicks in commercial production are highly sensitive to enteric infections and their resistance can be increased by administration of complex adult microbiota. However, it is not known which adult microbiota members are capable of colonising the caecum of newly hatched chicks. In this study, we therefore orally inoculated chicks with pure cultures of 76 different bacterial isolates originating from chicken caecum on day 1 of life and determined their ability to colonise seven days later. The caecum of newly hatched chickens could be colonised by bacteria belonging to phyla Bacteroidetes, Proteobacteria, Synergistetes, or Verrucomicrobia, and isolates from class Negativicutes (phylum Firmicutes). On the other hand, we did not record colonisation with isolates from phyla Actinobacteria and Firmicutes (except for Negativicutes), including isolates from families Lachnospiraceae, Ruminococcaceae, Erysipelotrichaceae, and Lactobacillaceae. Representatives of genera commonly used in probiotics such as *Lactobacillus*, *Enterococcus*, or *Bacillus* therefore did not colonise the chicken intestinal tract after a single dose administration. Following challenge with *Salmonella enterica* serovar Enteritidis, the best protecting isolates increased the chicken’s resistance to *S. Enteritidis* only tenfold, which, however, means that none of the tested individual bacterial isolates on their own efficiently protected chicks against *S. Enteritidis*.

## 1. Introduction

In animal species where parents raise their offspring, parents alone act as an important source of gut microbiota. This is also the case for chickens, who evolved for millions of years to be hatched in a nest in contact with a hen. However, contact between hen and chicks has been interrupted in commercial production, and the intestinal tract of commercially hatched chicks is gradually colonised from environmental sources only [1,2,3,4]. However, if the chicks are provided microbiota from a hen experimentally, they can be colonised by adult-type microbiota from the very first days of life [5,6].

The specific development of chick gut microbiota in commercial production is a reason why commercially hatched chicks are highly sensitive to infections, such as pathogenic *Escherichia coli*, *Clostridium perfringens*, or *Salmonella*, while chicks inoculated with faecal microbiota of adult hens are resistant to these infections [5,7,8,9]. This phenomenon, known as competitive exclusion, is well established in chickens [10]. However, administration of complex competitive exclusion microbiota from adult hens to newly hatched chickens, though effective in preventing infections, is not widely accepted. The reason is that such products may contribute to the spread of yet unknown pathogens. In agreement, we have shown that representatives of Campylobacteriales are present at a higher abundance in the chicks inoculated with the caecal content of donor hens than in the microbiota of the donor hen itself [6]. This issue can be avoided if products consisting of defined bacterial strains were used [11]. In poultry, such products contain mostly *Lactobacilli*, *Enterococci*, or *Bacilli* [12,13,14], although there are reports questioning their real protective effect against infections [15,16]. Whether this is due to the low microbial complexity of products consisting of one or a few strains or whether this is caused by inappropriate strain selection is not known.

Repeatedly confirmed efficacy of competitive exclusion products together with accumulating knowledge on chicken microbiota composition led us to systematic culture of individual chicken gut microbiota members [17,18]. Because previous studies indicated that not all bacterial species present in the faeces of adult hens efficiently colonise the chicken caecum [5,6], in this study, we tested pure cultures of 76 chicken gut anaerobes for their ability to colonise the caecum of newly hatched chicks. Using this approach, we addressed whether the tested bacterial isolates could (i) efficiently colonise the chicken caecum during the first week of life, and (ii) protect chicks against *Salmonella enterica* serovar Enteritidis infection. Conclusions were rather unexpected. The caecum of newly hatched chickens could be populated only with Gram-negative bacteria belonging to phyla Bacteroidetes, Proteobacteria, Synergistetes, or Verrucomicrobia, and Gram-positive isolates from class Negativicutes. On the other hand, chickens could not be colonised experimentally with Gram-positive isolates from phyla Actinobacteria or Firmicutes (except for those from class Negativicutes), including *Lactobacilli*, *Enterococci*, and *Bacilli*, which are commonly used as probiotics in chickens [12,13,14].

## 2. Materials and Methods

### 2.1. Ethics Statement

The handling of animals in the study was performed in accordance with current Czech legislation (Animal Protection and Welfare Act No. 246/1992 Coll. of the Government of the Czech Republic). The specific experiments were approved by the Ethics Committee of the Veterinary Research Institute followed by the Committee for Animal Welfare of the Ministry of Agriculture of the Czech Republic on 31 March 2016 (permit number 4/2016) and on 15 January 2018 (permit number MZe1922).

### 2.2. Bacterial Isolates

Seventy-six chicken gut anaerobes characterised previously [17] were used for oral inoculation of chicks on the day of hatching. Of these, 54 were used for oral inoculation of chickens as individual cultures. An additional 15 isolates were tested both as individual cultures and as a part of defined mixtures, and the remaining 7 isolates were tested only as a part of defined mixtures (Table S1).

### 2.3. Chicken Inoculation with Gut Anaerobes and S. Enteritidis Challenge

In all experiments, newly hatched male ISA Brown chicks were obtained from a local hatchery on the day of hatching. In total, 542 chicks were used in this study. Of these, 474 were inoculated with one of the tested isolates or their mixtures, and 68 chicks served as noninoculated controls across the whole study. The whole study was accomplished in 10 different experimental batches performed from 2016 to 2018 (Table S2). Chicks were reared in plastic boxes with free access to water and feed in air-conditioned rooms with a controlled light and temperature regime and with filtered air supply. Temperature was set to 30 °C during the first week of life and to 28 °C in the second week of life. The light regime was set to 24 h light in the first week of life and 22 h of light during the second week of life. The same standard starter feed formula for raising chickens during the first days of life was provided to chicks in all experiments (Table S3).

Chicks in experimental groups were orally inoculated on day 1 of life with 100 µL of a particular anaerobe (approx. 10^7^ CFU) resuspended in prereduced anaerobically sterilised (PRAS) solution to OD_600nm_ = 1 (PRAS solution composition - 0.1 g magnesium sulfate heptahydrate, 0.2 g monobasic potassium phosphate, 0.2 g potassium chloride, 1.15 g dibasic sodium phosphate, 3.0 g sodium chloride, 1.0 g sodium thioglycolate, 0.5 g L-cysteine, 1000 mL distilled water; final pH 7.5 +/− 0.2 at 25 °C). The cultures were washed from Wilkins–Chalgren agar (WCHA) after 3 days of incubation under anaerobic conditions at 37 °C as described previously [17]. Chicks in control groups were kept under the same conditions, but in separate rooms and without any treatment on day 1. On day 8 of life, half of the chicks in each group were euthanized, and caecal contents were frozen at −20 °C to examine caecal microbiota composition. In addition, sections of caecal tissue were collected in RNALater (Qiagen, Hilden, Germany) and stored at −80 °C prior to RNA purification. The remaining chicks were orally infected with *S. Enteritidis*, and the experiment was terminated 4 days postinfection.

### 2.4. Oral Inoculation with Defined Mixtures of Gut Anaerobes

In addition, selected gut anaerobes were also tested in mixtures. The isolates were grown separately and were mixed in an anaerobic cabinet Bactron600 (Sheldon Manufacturing Inc., Cornelius, OR, USA) to form the inoculum just prior to administration. In the first experiment, the chicks were inoculated with a mixture of bacteria cultured for 10 days (instead of the routine 3-day culture), testing whether stationary phase cells or spore-enriched cultures might affect their ability to colonise (see Table S1 for the composition of L10 and C10 mixtures). Next, we tested whether incubation of spore forming bacteria under nutrient-limited conditions might increase their potential to colonise chicks. Therefore, seven isolates were grown for 3 days on WCHA agar and thereafter resuspended either in sterile distilled water or sterile PRAS solution for 2 days before mixing and using as inoculum (Clost mixture in Table S1). The rest of these experiments were performed exactly as described above for individual isolates, i.e., newly hatched chicks were inoculated on day 1 of life with the mixture, half of the inoculated chicks were euthanised on day 8 to check for colonisation, and the second half of chicks was challenged with *S. Enteritidis*. Four days later, i.e., when the chicks were 12 days old, the experiment was terminated, caecal contents were collected, and *Salmonella* counts were determined.

In the last experiment with defined mixtures, we tested whether inoculation of chicks older than one day and initially precolonised with mostly Gram-negative bacteria might enable Gram-positive isolates to successfully colonise. The chicks were therefore inoculated with a mixture designated as BVL (Table S1) on day 1, followed by Clost mixture on day 8 of life.

### 2.5. Salmonella Enteritidis Challenge

*S. Enteritidis* challenge was performed orally with 1 × 10^7^ CFU *S. Enteritidis* 147 spontaneously resistant to nalidixic acid in 0.1 mL inoculum [19]. Four days after infection, the chicks were euthanised under chloroform anesthesia by cervical dislocation, and during necropsy, 0.5 g of caecum was collected to enumerate *S. Enteritidis*. In addition, sections of caecal tissue were collected in RNALater (Qiagen, Hilden, Germany) and stored at −80 °C prior to RNA purification.

### 2.6. Chicken Gene Expression

The chicken inflammatory response in the caecum was determined by quantitative reverse transcribed PCR (Qiagen, Hilden, Germany) quantifying the expression of extracellular fatty acid binding protein (ExFABP) as described previously [5]. This gene was selected due to its high basal expression in the caecum of chickens and also high induction following *S*. Enteritidis challenge [20].

### 2.7. Sequencing of V3/V4 Region of 16S rRNA Genes

Caecal contents were homogenised in a MagNALyzer (Roche, Prague, Czech Republic). The DNA was then extracted using a QIAamp DNA Stool Mini Kit according to the manufacturer’s instructions (Qiagen, Hilden, Germany), and the DNA concentration was determined spectrophotometrically. DNA samples were diluted to 5 ng/mL and were used as a template in PCR with forward primer 5′- *TCGTCGGCAGCGTCAGATGTGTATAAGAGACAG*-MID-GT-cctacgggnggcwgcag-3′ and reverse primer 5′-*GTCTCGTGGGCTCGGAGATGTGTATAAGAGACAG*-MID-GT gactachvgggtatctaatcc-3′.

The sequences in italics served for index and adapter ligation whereas the sequences in low case letters allowed the amplification over the V3/V4 region of 16S rRNA genes. MIDs represent different sequences of 5, 6, 7, or 9 base pairs in length, which were used to identify individual samples within the sequencing groups. PCR amplification was performed using a HotStarTaq Plus MasterMix kit (Qiagen, Hilden, Germany), and the resulting PCR products were purified using AMPure beads (Beckman Coulter, Prague, Czech Republic). In the next step, the concentration of PCR products was determined spectrophotometrically, the DNA was diluted to 100 ng/µL, and groups of 14 PCR products with different MID sequences were indexed with the same indices using Nextera XT Index Kit following the manufacturer’s instructions (Illumina, Cambridge, UK). Prior to sequencing, the concentration of differently indexed samples was determined using a KAPA Library Quantification Complete kit (Kapa Biosystems, Boston, MA, USA). All indexed samples were diluted to 4 ng/µL, and 20 pM phiX DNA was added to the final concentration of 5% (v/v). Sequencing was performed using MiSeq Reagent Kit v3 and MiSeq apparatus according to the manufacturer’s instructions (Illumina, Cambridge, UK).

Quality trimming of the raw reads was performed using TrimmomaticPE v0.32 [21] with the following parameters: Window size of 4 with 15 as average quality and minimal reads length 150 bp. The FASTQ files generated after quality trimming were uploaded into QIIME software [22]. Forward and reverse sequences were joined with minimum 8 bp overlap. In the next step, chimeric sequences were predicted by the slayer algorithm implemented in QIIME and excluded from subsequent analysis. The resulting sequences were then classified by RDP Seqmatch with an OTU (operational taxonomic units) discrimination level set to 97%. Principal coordinate analysis (PCoA) implemented in QIIME was used for data visualisation.

### 2.8. Statistics

To identify to which OTU an anaerobe used for inoculation belonged, a set of 76 “artificial” samples, each containing a single sequence of an anaerobe used for the inoculation, was included in the QIIME calculation. Partial 16S rRNA sequences of each anaerobe were thus assigned to particular OTUs, and the OTU abundance was compared in inoculated and control chickens by the nonparametric Mann–Whitney U test. In addition to statistical significance, differentially abundant OTUs must have been present in at least 0.1% average abundance in the microbiota of inoculated chicks, and the difference in their abundance in inoculated and control chicks must have been 10-fold or higher. The inflammatory response to colonisation with the tested anaerobes and to *S. Enteritidis* infection in inoculated and control chicks was evaluated by a Kruskal–Wallis test, followed by Dunn’s post hoc test. The likelihood of the ability to colonise and protect against *S. Enteritidis* challenge was tested by Chi2-test. In all cases, comparisons with *p* values lower than 0.05 were considered significant.

## 3. Results

### 3.1. Inoculation of Newly Hatched Chickens with Individual Bacterial Isolates

**Figure 1:**
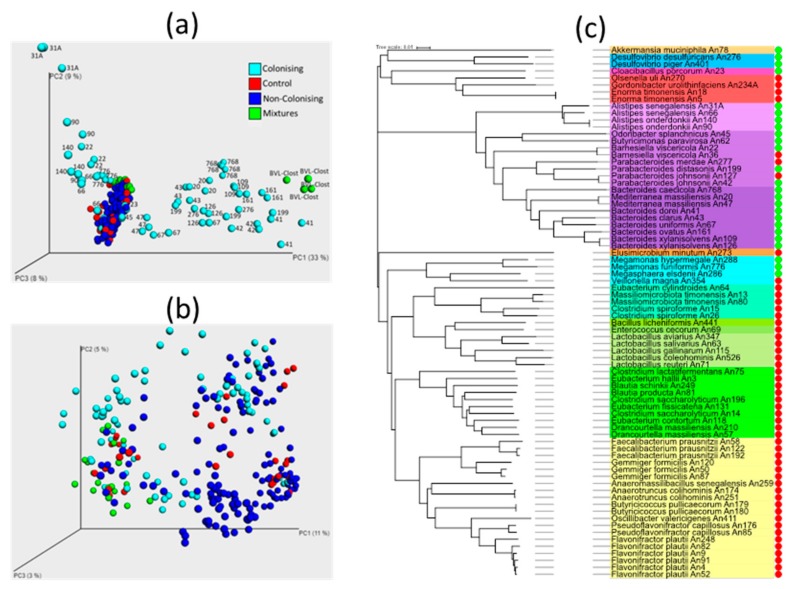
Gut microbiota composition in the caecum of newly hatched chicks. (**a**) Weighted principal coordinate analysis (PCoA) indicating gut anaerobes capable of colonisation. Chicks inoculated with isolates not capable of colonisation (dark blue dots) clustered together with control chicks (red dots). Dots outside this cluster represent chicks that were inoculated with isolates capable of caecum colonisation (light blue dots), and these are identified by the numbers of their An codes (see Table S1). Green dots—chicks inoculated with different defined mixtures. Not all isolates capable of colonisation can be seen in panel (**a**) because the projection of some successfully colonised chicks along PC3 resulted in an overlap with the cluster of control chicks. (**b**) According to unweighted PCoA, successful colonisation did not affect composition of the rest of the microbiota. The same colour coding is used in panels (**a**–**c**). Cluster alignment based on the whole gene sequence of 16S rRNA genes and ability to colonise the chicken caecum during the first week of life. Isolates with a green dot to the right of the dendrogram were detected in caeca of 8-day-old chicks. On the other hand, those with red dots were not detected. Families with a yellow, green, or light-blue background belong to phylum Firmicutes. Families with different shades of magenta belong to phylum Bacteroidetes.

Altogether, 76 isolates were used for oral inoculation of chicks on day 1 of life. Using PCoA for visualisation of gut microbiota composition in inoculated and control chicks on day 8 of life, the majority of inoculated chicks clustered together with noninoculated controls, i.e., isolates used for their inoculation likely did not colonise (Figure 1A). When the abundance of particular OTU representing the isolates used for oral inoculation was compared in inoculated and control chickens by the Mann–Whitney U test, only 25 isolates were capable of colonisation of the chicken caecum during the first week of life. These included 18 isolates belonging to phylum Bacteroidetes, 3 isolates belonging to phylum Firmicutes class Negativicutes, two isolates of Desulfovibrio (phylum Proteobacteria), and Akkermansia and Cloacibacillus belonging to phyla Verrucomicrobia and Synergistetes, respectively (Table 1). Except for Negativicutes, we did not record colonisation of chicks seven days after inoculation with any isolate belonging to phylum Firmicutes and the families Lactobacillaceae, Lachnospiraceae, Ruminococcaceae, or Erysipelotrichaceae.

Although we tested each bacterial isolate only in 3–5 chicks, altogether, 59 out of 69 chicks inoculated with isolates belonging to phylum Bacteroidetes were efficiently colonised, while not a single chick out of 110 inoculated with any isolate belonging to phylum Firmicutes was colonised (except for those belonging to class Negativicutes). We also failed with successful colonisation of the caecum of newly hatched chicks with four tested isolates belonging to phylum Actinobacteria (Figure 1C). Unweighted PCoA analysis showed that successful colonisation was of a low effect on the composition of other microbiota members as colonised, noncolonised, and control chicks were randomly distributed in the plot (Figure 1B). The apparent separation of some noncolonised chicks from colonised chicks was caused by minor experiment-to-experiment variation, similar to a previous report [23] (Figure S1).

The ability to colonise ranged among tested isolates. The most efficient colonisers (Alistipes senegalensis An31A, Bacteroides caecicola An768, Bacteroides dorei An41, Barnesiella viscericola An22, and Megamonas funiformis An776) formed over 50% of total caecal microbiota on day 8 of age. The least effective colonisers (Odoribacter splanchnicus An45, Butyricimonas paravirosa An62, Megasphaera elsdenii An286, Desulfovibrio desulfuricans An276, and Cloacibacillus porcorum An23) formed less than 1% of total microbiota, but this was still significantly more than in control chicks (Table 1, Table S4).

### 3.2. Additional Experiments to Test (In)Ability of Gram Positive Bacteria to Colonise The Chicken Caecum

The failure of any isolate from families Lactobacillaceae, Erysipelotrichaceae, Lachnospiraceae, and Ruminococcaceae to colonise chicken caecum after experimental administration was quite unexpected because these bacterial strains are common gut microbiota members and are among the first colonisers of the chicken caecum [3]. We considered that either the life cycle of these bacterial species required ingestion of spores rather than vegetative cells, or conditions in the caecum of chicks on the day of hatching, e.g., presence of residual oxygen, were not permissive for their colonisation. The former possibility was tested in two different experiments. In the first experiment, L10 and C10 mixtures of 10-day-old cultures (instead of 3-day-old cultures used in all previous experiments), enriched for stationary phase cells and spores, were used for the inoculation of newly hatched chicks (see Table S1 for the composition of L10 and C10 mixtures). Analysis of caecal microbiota seven days later showed that chicks inoculated with L10 or C10 mixture were not colonised by any isolate present in these mixtures. In the second experiment, Clost mixture was prepared by resuspension and incubation of individual isolates in nutrient-limited PRAS solution or in water for two days at room temperature in an anaerobic cabinet (see Table S1 for the composition of Clost mixture). However, even after such treatment, none of the seven isolates present in the Clost mixture colonised the chicken caecum.

To test the possibility that the caecum of newly hatched chicks was not permissive for Clostridiales due to the presence of residual oxygen, the chicks were first inoculated with BVL mixture containing bacteria mostly capable of colonisation (see Table S1 for the composition of BVL mixture), followed by inoculation with Clost mixture on day 8 of life. However, even in this experiment, none of the isolates present in the Clost mixture colonised the chicken caecum when checked on day 15 of life.

### 3.3. Chicken Response to Colonisation with Tested Anaerobes

**Figure 2:**
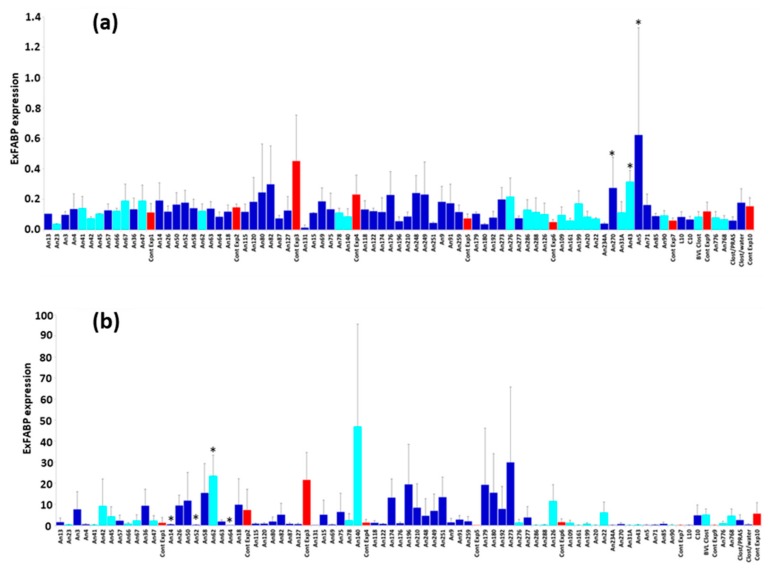
Extracellular fatty acid binding protein (ExFABP) expression in the caecum of inoculated and challenged chickens. (**a**) ExFABP expression in the caecum of 8-day-old chicks inoculated with different anaerobes on day 1 of life. Groups of chicks were inoculated on the day of hatching, and seven days later, expression of ExFABP was determined in the caecum of inoculated chicks by quantitative RT-PCR. Light-blue columns: ExFABP expression in the chicks that were successfully colonised by indicated gut anaerobes. Dark blue columns: ExFABP expression in the chicks that were inoculated with anaerobes that did not colonise the chicken caecum. Red columns highlight ExFABP expression in control chicks included in each experiment batch (except for the experiment with L10 and C10 mixtures in which no noninoculated chicks were included). *—Significantly different expression from appropriate control (Cont Exp7) by Kruskal–Wallis test followed by Dunn’s post hoc test (*p* < 0.05). (**b**) ExFABP expression in the caecum of 12-day-old chicks inoculated with different anaerobes on day 1 of life and infected with *S*. Enteritidis on day 8. If the isolate used for inoculation on day 1 exhibited a protective effect, lower expression was expected in the colonised chicks compared to the controls. Light-blue columns: ExFABP expression in the chicks that were successfully colonised by the tested isolate. Dark blue columns: ExFABP expression in the chicks that were inoculated by isolates that did not colonise the chicken caecum. Red columns: ExFABP expression in noninoculated control chicks included in each experiment batch (except for the experiment with L10 and C10 mixtures in which no noninoculated control chicks were included). *—Significantly different expression from the appropriate control (Cont Exp2) by Kruskal–Wallis test followed by Dunn’s post hoc test (*p* < 0.05).

If the tested isolates were commensals with probiotic potential, these should not have induced an inflammatory response characterised by increased expression of ExFABP [20]. Although ExFABP expression varied among the groups inoculated with different anaerobes, it was never induced to the levels induced by *S. Enteritidis* (compare *y*-axis scaling in Figure 2A,B). Despite this, significantly higher ExFABP expression than in control chicks was recorded in the chicks inoculated with *Bacteroides clarus* An43, *Olsenella uli* An270, and *Enorma timonensis* An5 (Figure 2A). However, because this happened in a single experiment and two of these species did not even colonise the chicken caecum, we concluded that the ExFABP expression was induced by other factor(s) and not by the isolates used for inoculation.

### 3.4. Chicken Response to S. Enteritidis Infection

Although *S. Enteritidis* infection increased ExFABP expression, there were only four significant differences in ExFABP expression between inoculated and control chickens (Figure 2B). Lower ExFABP expression in response to *S. Enteritidis* infection than in control chicks was recorded in chicks inoculated with [*Clostridium*] *saccharolyticum* An14, *Flavonifractor plautii* An52, or [*Eubacterium*] *cylindroides* An64. However, none of these anaerobes efficiently colonised chicken caecum. On the other hand, ExFABP expression in *Butyricimonas paravirosa* An62-colonised and *S*. Enteritidis-challenged chicks was significantly higher than in controls (Figure 2B). Because *Butyricimonas paravirosa* An62 was present in the caecum at the time of infection, this bacterium therefore did not protect chickens against *S. Enteritidis* infection and corresponding inflammatory response.

**Figure 3:**
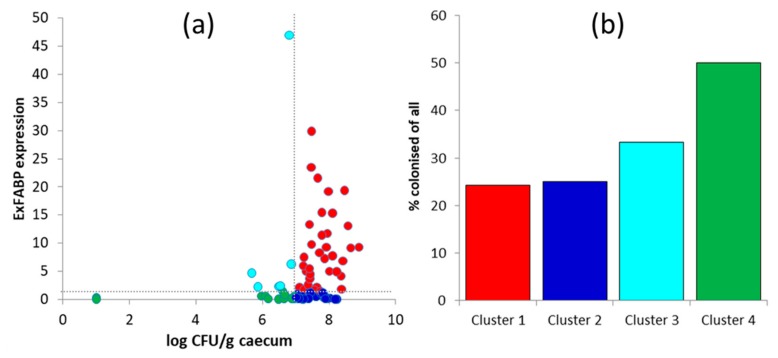
*Salmonella* counts and chicken inflammatory response. (**a**) Each dot represents a group of chickens inoculated with a particular anaerobe whose position is defined by average *S*. Enteritidis counts (log_10_ CFU/g) and inflammatory response determined by ExFABP expression. Cluster 1 (red dots) is formed by the chickens that were inoculated with gut anaerobes that did not protect them against *S*. Enteritidis challenge (high *S*. Enteritidis count and high ExFABP expression). Cluster 2 (dark blue dots) comprised chicks that were inoculated with gut anaerobes that did not protect them against *S*. Enteritidis colonisation, but decreased chicken inflammatory response. Chickens in cluster 3 (light blue dots) were inoculated with gut anaerobes, which protected them against *S*. Enteritidis colonisation, but did not decrease the chicken inflammatory response. Chickens in cluster 4 (green dots) were inoculated with gut anaerobes, which increased their resistance to *S. Enteritidis* colonisation without an excessive inflammatory response. Panel (**b**) represents the percentage of efficiently colonised chicks out of all inoculated belonging to clusters 1–4 defined in panel (**a**). The same colour coding in panels (**a**,**b**) is used.

Evaluation of *S. Enteritidis* counts in inoculated and control chicks was complicated by the fact that 3–5 chicks per group were challenged, and in some cases, other bacterial species overgrew *S*. Enteritidis on Xylose-Lysine-Deoxycholate (XLD) plates (Figure S2). To characterise the interaction of *S*. Enteritidis, chicken host, and gut microbiota, we therefore combined *S. Enteritidis* counts in the caecum and expression of ExFABP. When these two parameters were plotted against each other, the groups of chicks were divided into those with *S*. Enteritidis counts higher or lower than 10^7^ CFU/g of caecal content and exhibiting a high or low inflammatory response according to ExFABP expression. A threshold value for ExFABP expression was defined as 10 times higher than average ExFABP expression prior to *S*. Enteritidis infection. This resulted in the formation of four different clusters (Figure 3A).

Cluster 1 comprised chicks with high *S*. Enteritidis counts and a high inflammatory response. These chicks were inoculated on day 1 of life with 37 different isolates, and only nine of them (24.32%) efficiently colonised the chicken caecum (Figure 3B). Cluster 2 comprised chicks with high *S*. Enteritidis counts, but a low inflammatory response. These chicks were inoculated with 24 different isolates, and six of them (*Bacteroides dorei* An41, *Bacteroides xylanisolvens* An109, *Desulfovibrio desulfuricans* An276, *Megamonas hypermegale* An288, *Megasphaera elsdenii* An286, and *Mediterranea massiliensis* An20) efficiently colonised the chicken caecum. Cluster 3 comprised chicks with low *S*. Enteritidis counts, but a high inflammatory response. These chicks were inoculated with six different isolates, and two of them (*Alistipes onderdonkii* An140, *Akkermansia muciniphila* An78) efficiently colonised the chicken caecum. Cluster 4 comprised chicks with low *S*. Enteritidis counts and a low inflammatory response. These chicks were inoculated with 16 different isolates, and eight of them (*Bacteroides ovatus* An161, *Bacteroides clarus* An43, Alistipes onderdonkii An90, *Alistipes senegalensis* An66, *Alistipes senegalensis* An31A, *Megamonas funiformis* An776, *Parabacteroides distasonis* An199, and *Cloacibacillus porcorum* An23) efficiently colonised the chicken caecum.

## 4. Discussion

In this study, we tested which bacterial isolates commonly found in chicken caecal microbiota could efficiently colonise the chicken caecum after an experimental, single dose administration on the day of hatching. Such information is important for understanding general principles of microbiota–host interactions and evidence-based design of next-generation probiotics.

We have shown that chickens can be colonised with bacteria expressing the outer membrane present in Gram-negative bacteria and isolates from class Negativicutes belonging to Gram-positive Firmicutes. All the remaining Gram-positive isolates did not colonise the chicken caecum during the first week of life. The ability of Gram-negative bacteria to colonise corroborates earlier findings that characterised the bacterial species colonising the chicken caecum after administration of caecal extracts [5,6,24] or after contact with an adult hen [25]. The isolates that successfully colonised the caeca of chicks did not require any other microbiota members to co-colonise. These colonised the chicken caecum independently and their colonisation was of little influence on the colonisation of other microbiota members. The rather small effect on other microbiota members can also be seen in their ability to affect *S. Enteritidis* colonisation. Though some of the isolates decreased *S. Enteritidis* counts by one log of magnitude, this reduction must be considered as minor because colonisation with complex microbiota reduces *Salmonella* counts by five logs [5].

The inability of isolates belonging to families Lactobacillaceae, Lachnospiraceae, Ruminococcaceae, and Erysipelotrichaceae to colonise the chicken caecum was quite unexpected as such isolates are common microbiota members and belong to the first colonisers of the chicken caecum in commercially hatched chicks [1,2,3]. Moreover, *Lactobacilli*, *Bacilli*, and *Enterococci* are used as probiotics, although their positive effect on chicken gut health has not been always confirmed [15,16]. Several explanations for their inability to colonise are possible, although none of them conclusively explain the recorded observations. It is possible that isolates of families Lachnospiraceae, Ruminococcaceae, and Erysipelotrichaceae have to enter the intestinal tract in the form of spores. When we addressed this, we did not record successful colonisation, although we cannot exclude that we did not meet the conditions required for spore formation of the tested isolates. It is also possible that conditions in the caeca of chicks during the first week of life are not permissive for the colonisation with Gram-positive bacteria. However, this contradicts epidemiological findings showing that Lachnospiraceae and Ruminococcaceae belong among the first colonisers [1,3,4]. Moreover, we also did not record Lachnospiraceae and Ruminococcaceae colonisation when these were administered to chicks already precolonised with *Bacteroides* species. We also considered that there could be a mutual dependence among bacterial species from families Lachnospiraceae, Ruminococcaceae, and Erysipelotrichaceae, but even when we inoculated these isolates in mixtures, we did not record efficient colonisation. Perhaps the time window allowing initial colonisation of Lachnospiraceae, Ruminococcaceae, or Erysipelotrichaceae is available earlier during the chicken life, shortly after hatching. This would be consistent with the fact that commercial, newly hatched chickens in fact represent a heterogeneous group of chicks that may differ in their age up to 48 h. However, we also consider the hypothesis that bacterial species from these families may not permanently colonise the chicken caecum and have to be continuously supplied from the environment.

No matter which of the explanations is/are correct, Gram-positive bacteria seem to be less suitable for probiotic products intended for a single dose administration, as these did not efficiently colonise chicken caecum under our experimental conditions. Instead, such products should consist mainly of Gram-negative bacteria, capable of efficient colonisation of chicken caecum, sources of which are limited in an environment. However, such products will have to consist of multiple carefully selected bacterial strains because individual strains only weakly protected chickens against pathogens like *Salmonella*.

## Figures and Tables

**Table 1:** List of isolates capable of colonising the chick caecum during the first week of life.

Species	ID	Phylum	% of Total Microbiota
*Alistipes onderdonkii*	An140	Bacteroidetes	34.24
*Alistipes onderdonkii*	An90	Bacteroidetes	33.54
*Alistipes senegalensis*	An31A	Bacteroidetes	78.03
*Alistipes senegalensis*	An66	Bacteroidetes	1.74
*Bacteroides caecicola*	An768	Bacteroidetes	63.01
*Bacteroides clarus*	An43	Bacteroidetes	29.92
*Bacteroides dorei*	An41	Bacteroidetes	52.51
*Bacteroides ovatus*	An161	Bacteroidetes	31.35
*Bacteroides uniformis*	An67	Bacteroidetes	23.85
*Bacteroides xylanisolvens*	An109	Bacteroidetes	13.09
*Bacteroides xylanisolvens*	An126	Bacteroidetes	4.96
*Odoribacter splanchnicus*	An45	Bacteroidetes	0.83
*Butyricimonas paravirosa*	An62	Bacteroidetes	0.72
*Barnesiella viscericola*	An22	Bacteroidetes	65.08
*Mediterranea massiliensis*	An20	Bacteroidetes	47.46
*Mediterranea massiliensis*	An47	Bacteroidetes	11.36
*Parabacteroides distasonis*	An199	Bacteroidetes	39.76
*Parabacteroides johnsonii*	An42	Bacteroidetes	25.18
*Megamonas funiformis*	An776	Firmicutes/Negativicutes	53.32
*Megamonas hypermegale*	An288	Firmicutes/Negativicutes	8.62
*Megasphaera elsdenii*	An286	Firmicutes/Negativicutes	0.98
*Desulfovibrio piger*	An401	Proteobacteria	1.64
*Desulfovibrio desulfuricans*	An276	Proteobacteria	0.65
*Akkermansia muciniphila*	An78	Verrucomicrobia	13.08
*Cloacibacillus porcorum*	An23	Synergistetes	0.25

## Supplementary Materials

The following are available online at https://www.mdpi.com/2076-2607/7/12/597/s1, Figure S1: Unweighted PCoA visualisation of gut microbiota composition in the caecum of newly hatched chicks. Figure S2: *Salmonella* counts in the caecum of 12-day-old chicks inoculated with different anaerobes on day 1 of life and challenged with *S*. Enteritidis on day 8. Table S1: OTU table of OTUs forming at least 0.05% of all microbiota in at least one of the tested samples. Table S2: List of strains used in this study. Table S3: Bacterial strains and numbers of chicks used in different experiments. Table S4: Feed composition, light, and temperature regime used during rearing.
