# Supplementary material for: Gut Anaerobes Capable of Chicken Caecum Colonisation
Source: Microorganisms. 2019 Nov 21;7(12):597. doi: 10.3390/microorganisms7120597 (PMC6956218; doi:10.3390/microorganisms7120597)

Figure S1. Unweighted PCoA visualisation of gut microbiota composition in the caecum of newly hatched chicks. Unweighted PCoA analysis showed that successful colonisation was of low effect on the composition of other microbiota members since inoculated and colonised, inoculated but not colonised, and control chicks were randomly distributed in the plot (upper panel). Apparent separation of some inoculated but non-colonised chicks from inoculated and colonised chicks was caused by minor experiment to experiment variation similar to previous report (lower panel with colour identification of chicks from the same experiment).

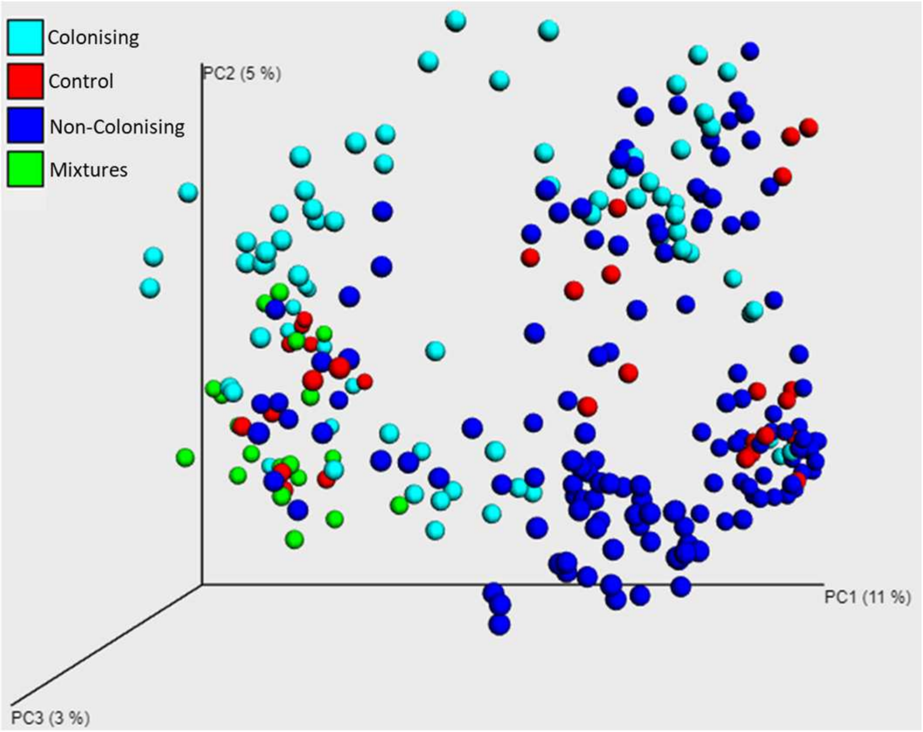

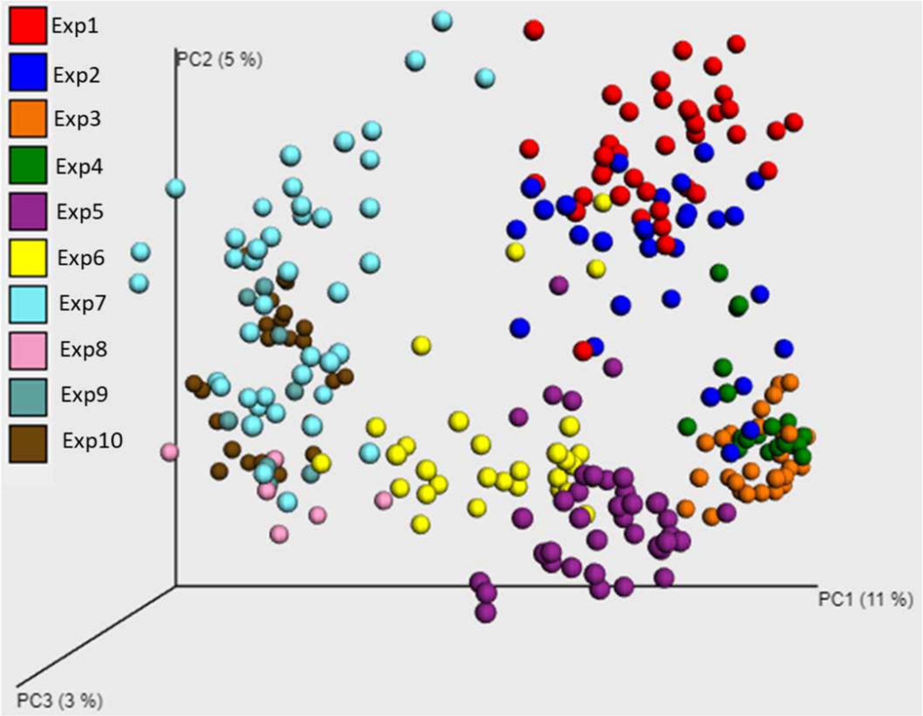

Supplement: Supplementary file 1 [file microorganisms-07-00597-s001.zip › Figure S1.pdf]
