# Supplementary material for: Gut Anaerobes Capable of Chicken Caecum Colonisation
Source: Microorganisms. 2019 Nov 21;7(12):597. doi: 10.3390/microorganisms7120597 (PMC6956218; doi:10.3390/microorganisms7120597)

Figure S2. *Salmonella* counts in the caecum of 12-day-old chicks inoculated with different anaerobes on day 1 of life and challenged with S. Enteritidis on day 8. Light blue columns, *Salmonella* counts in the chicks which were successfully colonised. Dark blue columns, *Salmonella* counts in the chicks which were inoculated but tested isolate did not colonise chicken caecum. Red columns highlight *Salmonella* counts in non-inoculated control chicks included in each experiment batch (except for the experiment with L10 and C10 mixtures in which no non-inoculated control chicks were included).

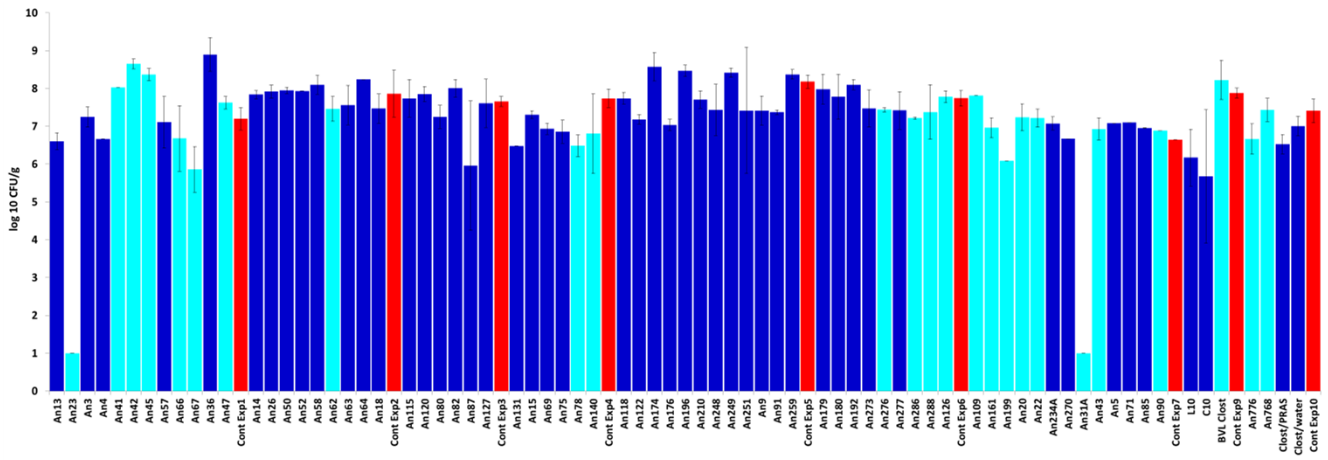

Supplement: Supplementary file 1 [file microorganisms-07-00597-s001.zip › Figure S2.pdf]
